# Endoscopic characteristics and clinical management of cap polyposis: Insights from seven cases

**DOI:** 10.1055/a-2870-7562

**Published:** 2026-06-08

**Authors:** Jifang Cui, Luyao Zhao, Muran Li, Xiuqiang Huang, Yandi Liu

**Affiliations:** 1 Department of Gastroenterology 74769 Tianjin Union Medical Center, The First Affiliated Hospital of Nankai UniversityTianjinChina

**Keywords:** cap polyposis, ulcerative colitis, *Helicobacter pylori*, treatment

## Abstract

**Background and study aims:**

Cap polyposis (CP) is an uncommon benign condition affecting the colorectal region. This study aimed to examine endoscopic features, therapeutic responses, and clinical outcomes of CP, with particular emphasis on the variable response to *Helicobacter pylori* eradication.

**Patients and methods:**

This retrospective case series evaluated seven patients diagnosed with CP at Tianjin Union Medical Center between February 2018 and April 2023. The diagnosis was established based on distinctive endoscopic and histological findings, with clinical exclusion of other conditions such as ulcerative colitis (UC) and solitary rectal ulcer syndrome. Clinical data, endoscopic characteristics, treatments, and outcomes were systematically reviewed.

**Results:**

This case series included seven patients (six males, one female) with a median age of 36 years. Four patients were *H. pylori* -positive. All patients exhibited characteristic sessile or semi-pedunculated polyps covered by a whitish fibrinopurulent cap, predominantly located in the rectosigmoid region with normal intervening mucosa. Treatment outcomes varied. Two *H. pylori* -positive patients with short disease duration achieved complete remission following eradication therapy combined with polypectomy. Two other *H. pylori* -positive patients showed only initial improvement after eradication, with subsequent recurrences; one ultimately required total colectomy due to disease progression. In contrast, one *H. pylori* -negative patient achieved remission through polypectomy and medical therapy alone.

**Conclusions:**

CP poses significant diagnostic challenges and demonstrates heterogeneous responses to treatment. Eradication of *H. pylori* may prove effective in certain patients with early-stage disease. Therefore, timely diagnosis and personalized treatment strategies, including endoscopic resection, are essential for achieving favorable outcomes.

## Introduction

Cap polyposis (CP), initially identified in 1985, is an uncommon disorder characterized by inflammatory polyps in the colorectum, with an increasing number of cases being documented in the literature 1 2 . This condition can affect individuals across a broad age spectrum, with some studies indicating a higher prevalence in males 3 . Clinically, CP presents with symptoms such as diarrhea, mucous discharge, rectal bleeding, and tenesmus. In severe instances, it may lead to anemia and hypoalbuminemia due to protein-losing enteropathy 4 . CP is often misdiagnosed as ulcerative colitis (UC) because of shared clinical and endoscopic features. Nevertheless, there are critical differences: UC is characterized by diffuse, continuous inflammation, whereas CP presents with discrete polypoid lesions located at the apices of transverse folds, with the intervening mucosa appearing normal upon endoscopic examination 5 . Polyps in CP have a distinctive endoscopic appearance, manifesting as sessile or semi-pedunculated lesions covered by a thick whitish exudate, forming a “cap” 3 6 . Histologically, UC is marked by chronic active inflammation with crypt distortion, whereas CP is distinguished by elongated, hyperplastic crypts covered by a “cap” of inflammatory granulation tissue and fibrinous exudate, with inflammation confined to the polyp and sparing the intervening mucosa 5 7 . This study provides a comprehensive analysis of seven cases of CP, emphasizing clinical presentation, endoscopic observations, and varied responses to treatment, with a particular focus on *Helicobacter pylori* .

## Patients and methods

Conducted as a retrospective case series, the study encompassed consecutive patients diagnosed with CP at the Department of Gastroenterology, Tianjin Union Medical Center, from February 2018 to April 2023. Diagnosis of CP was confirmed based on distinctive endoscopic characteristics, including discrete polyps located at the apices of transverse folds, covered by a whitish cap, with normal intervening colorectal mucosa and corroborated by histopathological findings, which included elongated crypts with a fibrinopurulent cap and inflamed granulation tissue. This diagnosis was made after excluding other inflammatory or neoplastic conditions, such as UC and solitary rectal ulcer syndrome (SRUS). Clinical data, encompassing demographics, symptoms, disease duration, endoscopic findings, *H. pylori* status, treatment modalities, and outcomes, were extracted from electronic medical records. The study protocol involving human participants received approval from the Ethics Committee of Tianjin Union Medical Center. Informed consent for the publication of anonymized data was obtained from all participants.

## Results

The analysis encompassed a cohort of seven patients diagnosed with CP, comprising six males and one female, with a median age of 36 years (range: 22–71 years). Among these patients, four (57.1%) tested positive for *H. pylori* . A comprehensive summary of patient demographics, clinical presentations, endoscopic findings, treatment regimens, and clinical outcomes is provided in Table 1 .

**Table:** **Table 1** Summary of seven patients with cap polyposis.

Patient	Age/gender	DCBC (y or mo)	Symptoms	Affected sites	Endoscopic findings	Complications	Treatment	Follow-up
1	71/F	13 y	Mucous and bloody stools, tenesmus, abdominal pain, edema legs, weight loss	Entire colon	Erythematous mucosa and polyps separated by white acanthoid mucosa	HP (+), Anemia, hypoalbuminemia	Polypectomy, mesalazine, *H. Pylori* eradication, metronidazole, steroid enema, total proctocolectomy	Progression
2	63/M	8 y	Mucous and bloody stools, tenesmus, abdominal pain, edema legs, weight loss	Descending colon, sigmoid colon, rectum	Erythematous mucosa and polyps separated by white acanthoid mucosa	Anemia, hypoalbuminemia, luminal obstruction	Polypectomy, mesalazine, metronidazole, steroid enema	Lost to follow-up
3	36/M	3y	Mucous and bloody stools, tenesmus, abdominal pain, edema legs, weight loss	Transverse colon, descending colon, sigmoid colon, rectum	Sessile reddish polyps	HP (+), Anemia, hypoalbuminemia, chronic myelogenous leukemia	Polypectomy, mesalazine, *H. Pylori* eradication, steroid enema, intravenous steroid	Progression
4	52/M	7 y	Mucoid, bloody diarrhea	Sigmoid colon, rectum	Sessile reddish polyps	HP (-)	Polypectomy, mesalazine	Recovery
5	22/M	1 mo	Rectal bleeding	Rectum, anus	Sessile reddish polyps	HP (+)	Polypectomy, mesalazine, *H. Pylori* eradication	Improvement
6	22/M	2 y	Mucoid, bloody diarrhea	Sigmoid colon, rectum	Sessile reddish and flat-topped protruding polys	Hypoalbuminemia, HP (+)	Polypectomy, mesalazine, *H. Pylori* eradication	Improvement
7	22/M	2 mo	Bloody stool	Rectum	Sessile reddish polyps	Anemia	Mesalazine, steroid enema	Recovery
DCBC, disease course before confirmation.								

### Patient 1

**Fig. 1:**
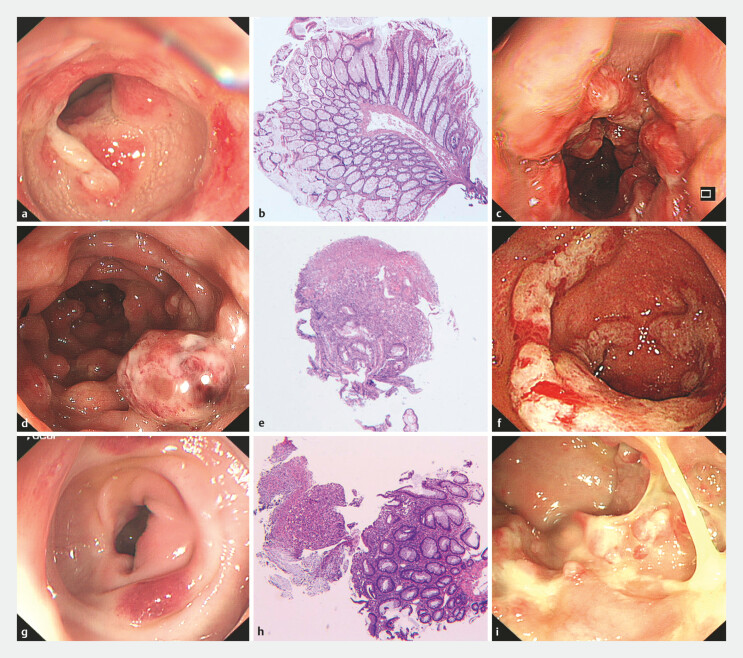
Endoscopic and histopathologic features of CP. **a** Patient 1: Endoscopy reveals erythematous mucosal patches interspersed with polyps and thickened white mucosa. **b** Patient 1: Histology demonstrates inflamed mucosa with elongated, tortuous crypts tapering toward the luminal surface, capped by superficial fibrinopurulent exudate. **c** Patient 2: Serial endoscopy documents progressive polyp enlargement and increased density, resulting in near-complete luminal obstruction. **d** Patient 3: Endoscopy identifies multiple inflammatory polyps at transverse mucosal fold apices. **e** Patient 3: Histology reveals focal surface ulceration with subepithelial granulation tissue. **f** Patient 6: Endoscopy demonstrates a characteristic flat-topped protruding polyp in the descending colon. **g** Patient 6: Follow-up colonoscopy at 4 months post-treatment reveals complete polyp resolution with residual erythematous mucosa. **h** Patient 6: Histology shows a polypoid lesion exhibiting mucosal hyperplasia, surface necroinflammatory debris, and underlying stromal granulation tissue. **i** Patient 7: Endoscopy visualizes fibrinopurulent exudate-covered polypoid lesions in the rectosigmoid colon.

A 71-year-old woman with a 13-year history of UC presented with recurrent mucoid, bloody diarrhea (2–5 episodes/day). Despite multiple hospitalizations and treatment with oral mesalazine and steroid enemas, symptoms persisted. Two months prior to referral, she developed exacerbated blood y stools (> 10 episodes/day) following hip replacement surgery and anticoagulant therapy. Initial colonoscopy revealed colitis and polyps, prompting polypectomy. Upon transfer to our institution, she exhibited leg edema, anemia (hemoglobin [Hb] 96 g/L), elevated C-reactive protein (CRP) (4.29 mg/L), and hypoalbuminemia (32.8 g/L). Computed tomography (CT) demonstrated diffuse colonic wall thickening. Repeat colonoscopy showed erythematous patches and polyps from the descending colon to the rectum, separated by white acanthoid mucosa ( Fig. 1 **a** ). A positive *H. pylori* test led to eradication therapy and steroid administration, yielding symptom resolution. Six months later, steroid treatment was gradually withdrawn and the patient experienced mucous and bloody stools again, more than 10 times per day. At this time, reexamination of colonoscopy and abdominal CT revealed that the lesion was narrowed to the sigmoid colon and rectum. After oral administration and enema of steroid again, the patient’s stool returned to normal.

Six months post-steroid tapering, symptoms recurred (> 10 bloody stools/day). Colonoscopy and CT localized disease to the sigmoid colon and rectum. Steroid reinitiation normalized stools. Four months later, she returned with severe weakness, 20 to 30 daily bloody stools, and hypoalbuminemia (26 g/L). Imaging confirmed pan-colonic inflammation, necessitating total colorectal resection. Histopathology confirmed CP ( Fig. 1 **b** ).

### Patient 2

A 63-year-old male with 8 years of intermittent diarrhea underwent eight polypectomies for rectosigmoid polyps, initially misdiagnosed as adenomatous polyps. Subsequent mucoid bloody stools (3 episodes/day) prompted colonoscopy, revealing erythematous, edematous rectosigmoid mucosa misattributed to UC. Mesalazine and steroid enemas transiently improved symptoms. *H. pylori* status was not assessed. One year prior, symptoms escalated (> 10 bloody stools/day, 15 kg weight loss), with hypoalbuminemia (29.9 g/L) and elevated CRP (76.08 mg/L). Colonoscopy demonstrated near-obstructive polyps extending to the descending colon ( Fig. 1 **c** ). Surgical intervention was advised but declined; the patient was lost to follow-up.

### Patient 3

A 36-year-old male with chronic myelogenous leukemia (CML) reported 3 years of mucoid bloody stools and abdominal pain. Anemia (Hb 115 g/L) and hypoalbuminemia (29.2 g/L) were noted. Colonoscopy identified fibrinopurulent exudate-covered polyps from the rectum to the transverse colon ( Fig. 1 **d, e** ). Mesalazine, steroid enemas, and *H. pylori* eradication reduced symptoms. Recurrence 5 months later responded to intravenous (IV) steroids, although polyps persisted.

### Patient 4

A 52-year-old male with 7 years of recurrent mucoid bloody stools exhibited rectosigmoid polyps at transverse fold apices. *H. pylori* testing was negative. Repeated polypectomy and mesalazine achieved symptomatic and endoscopic remission without the need for eradication therapy.

### Patient 5

A 22-year-old male presented with a 1-month history of rectal bleeding. Colonoscopic examination identified sessile reddish polyps located in the rectum and anus and the patient tested positive for *H. pylori* . Following polypectomy, mesalazine therapy, and *H. pylori* eradication, the patient exhibited clinical improvement.

### Patient 6

A 22-year-old male with a 2-year history of mucoid bloody diarrhea presented with sessile reddish and flat-topped protruding polyps in the sigmoid colon and rectum. The patient tested positive for *H. pylori* and exhibited hypoalbuminemia. He underwent polypectomy, mesalazine therapy, and *H. pylori* eradication, which resulted in clinical improvement ( Fig. 1 **f-h** ).

### Patient 7

A 22-year-old male presented with a 2-month history of bloody stools. Colonoscopy revealed sessile reddish polyps in the rectum; however, the patient's *H. pylori* status was not assessed. Treatment with mesalazine and steroid enema led to complete recovery ( Fig. 1 **i** ).

## Discussion

Etiology of CP remains incompletely elucidated, with several hypothesized mechanisms including mucosal prolapse, chronic inflammation, and infectious triggers 1 3 . Histologically, CP exhibits characteristics akin to mucosal prolapse syndrome, such as fibromuscular obliteration and smooth muscle proliferation within the lamina propria, suggesting that mechanical stress resulting from abnormal colonic motility or chronic straining may contribute to its development 3 5 . Nevertheless, the inconsistent response to conservative interventions, such as avoidance of straining, implies involvement of additional factors 1 .

The association between *H. pylori* infection and CP in our cohort reveals significant variability. Among the seven patients studied, four (Patients 1, 3, 5, and 6) tested positive for *H. pylori* and underwent eradication therapy. Notably, Patients 5 and 6, both young males with relatively short disease duration and limited polyp burden, achieved complete clinical and endoscopic remission following eradication therapy in conjunction with polypectomy. Conversely, Patients 1 and 3 experienced only initial improvement post-eradication, with subsequent recurrences necessitating further interventions. In the present study, Patient 1, an elderly individual with a prolonged history of UC and extensive colonic involvement, ultimately required a total colectomy. Patient 3, who had comorbid CML, exhibited persistent polyps despite treatment with IV steroids. Patient 4, who tested negative for *H. pylori* , achieved remission through polypectomy and mesalazine monotherapy. In contrast, Patients 2 and 7, who were not assessed for *H. pylori* infection, experienced divergent clinical outcomes: Patient 2 showed disease progression despite multiple therapeutic interventions, whereas Patient 7 achieved recovery with administration of mesalazine and steroid enemas. This variability in treatment response suggests that effectiveness of *H. pylori* eradication in chronic polyposis may be influenced by several factors, including patient age, disease duration, extent of colonic involvement, and presence of comorbid conditions. Early-stage disease characterized by a limited polyp burden appears to predict more favorable response to eradication therapy, as demonstrated by Patients 5 and 6. Conversely, advanced disease with extensive colonic involvement or complicating comorbidities may necessitate more aggressive treatment strategies, irrespective of *H. pylori* status.

Mechanisms through which *H. pylori* infection may contribute to CP remain speculative. Given that *H. pylori* has not been directly identified within colonic polyp tissue 2 , indirect mechanisms have been proposed. Persistent *H. pylori* infection might induce a systemic immune response or chronic immune stimulation, potentially resulting in production of autoantibodies or cross-reactivity between *H. pylori* antigens and colonic epithelial components 8 . Alternatively, antibiotics used in eradication therapy might target other, yet unidentified bacterial pathogens that contribute to CP pathogenesis. The hypothesis of gut microbiota dysbiosis has gained support, with studies indicating significant alterations in fecal microbiota composition following antibiotic treatment in CP patients 9 . The 2016 revised Japanese guidelines for *H. pylori* management categorize CP as a condition “presumed to be associated with *H. pylori* infection” suggesting that eradication therapy may be considered for affected patients 10 . Our findings support this nuanced approach: Although *H. pylori* eradication can be effective in selected patients, particularly those with early-stage disease and limited polyp burden, it is not uniformly successful. Consequently, testing for and eradication of *H. pylori* should be integrated into a comprehensive management strategy. However, clinicians must maintain realistic expectations and be prepared to explore additional therapeutic approaches if response is insufficient.

CP is often misdiagnosed as UC 5 11 . In this study, we recognize that the systematic performance of background mucosal biopsies was not conducted in this retrospective series, a limitation that we now explicitly acknowledge. Nevertheless, diagnosis of CP was substantiated by characteristic endoscopic findings, such as polyps located at the apices of folds with normal intervening mucosa and a whitish exudative “cap” as well as histological features, including fibrinopurulent caps without diffuse crypt distortion, and the clinical course, which did not progress into diffuse colitis. The literature underscores that the critical differentiation lies in the intervening mucosa: In CP, it remains normal, whereas in UC, even areas that appear normal often exhibit microscopic chronic inflammation 3 5 . Furthermore, CP polyps are characterized by a distinctive “cap” which is not typical of UC-associated polyps 5 7 . In elderly patients (Patients 1 and 2), SRUS was considered due to overlapping histological features. In contrast to the typical presentation of SRUS as a solitary lesion on the anterior rectal wall with submucosal fibromuscular obliteration, our patients exhibited multiple polyps along the transverse folds with alterations confined to the mucosa 11 . However, it is important to recognize that SRUS continues to be a relevant factor in elderly patients presenting with prolapse-type histology. We have underscored the necessity of acquiring a comprehensive defecatory history in these cases.

Currently, there are no standardized therapeutic guidelines for CP. Although eradication of *H. pylori* may induce remission in some cases 2 8 , endoscopic polypectomy is frequently required. Both endoscopic mucosal resection and endoscopic submucosal dissection are considered safe and effective, providing complete resection with low complication rates 6 . Nevertheless, recurrence rates remain high, particularly in cases with extensive involvement. Factors contributing to recurrence include polyp multiplicity, circumferential growth, severe mucosal prolapse, refractory chronic inflammation, and delayed diagnosis or intervention 3 . For lesions located near the dentate line or in refractory cases, surgical resection may be necessary, although the possibility of recurrence persists 3 6 .

This case series highlights the considerable variability in disease progression among patients. Elderly Patients 1 and 2, both of whom experienced prolonged symptoms and a delayed definitive diagnosis, exhibited disease progression despite undergoing multiple treatment modalities. Patient 3, whose treatment was also delayed due to comorbid leukemia, demonstrated persistent disease. Conversely, younger Patients 4 through 7, who received timely interventions, achieved remission. Notably, among the *H. pylori* -positive cohort, those who were treated early in their disease course (Patients 5 and 6) exhibited more favorable outcomes compared with those with extended disease duration (Patients 1 and 3). These findings suggest that early diagnosis and individualized treatment are critical in preventing disease progression. Our observations are consistent with existing literature, which indicates that younger patients with a shorter disease duration tend to experience better outcomes 3 .

## Conclusions

In summary, CP poses significant diagnostic and therapeutic challenges. Precise differentiation from UC and SRUS necessitates comprehensive assessment incorporating endoscopic, histological, and clinical evaluations. Endoscopic observation of a whitish “cap” over sessile polyps in the rectosigmoid region, with intervening normal mucosa, should prompt consideration of CP. Testing for *H. pylori* is advisable, given potential for remission in certain patients, especially those with early-stage disease. Nevertheless, treatment responses can vary, and endoscopic resection remains the primary approach for managing localized disease. Prompt recognition and tailored treatment strategies are essential for achieving favorable patient outcomes.
